# Medical error disclosure: from the therapeutic alliance to risk management: the vision of the new Italian code of medical ethics

**DOI:** 10.1186/1472-6939-15-57

**Published:** 2014-07-15

**Authors:** Emanuela Turillazzi, Margherita Neri

**Affiliations:** 1 Department of Legal Medicine, University of Foggia, Via degli Aviatori, 1, 71100 Foggia, Italy

**Keywords:** Duty of information, Error disclosure, Italian code of medical ethics, Patients’ safety, Risk management

## Abstract

**Background:**

The Italian code of medical deontology recently approved stipulates that physicians have the duty to inform the patient of each unwanted event and its causes, and to identify, report and evaluate adverse events and errors. Thus the obligation to supply information continues to widen, in some way extending beyond the doctor-patient relationship to become an essential tool for improving the quality of professional services.

**Discussion:**

The new deontological precepts intersect two areas in which the figure of the physician is paramount. On the one hand is the need for maximum integrity towards the patient, in the name of the doctor’s own, and the other’s (the patient’s) dignity and liberty; on the other is the physician’s developing role in the strategies of the health system to achieve efficacy, quality, reliability and efficiency, to reduce errors and adverse events and to manage clinical risk.

**Summary:**

In Italy, due to guidelines issued by the Ministry of Health and to the new code of medical deontology, the role of physicians becomes a part of a complex strategy of risk management based on a system focused approach in which increasing transparency regarding adverse outcomes and full disclosure of health- related negative events represent a key factor.

## Background

Recent developments in the international cultural debate have brought to the fore, in Italy, the question of the so-called ‘duty of candour’ of physicians towards patients [1]. The topic arises at a complex time in which a long series of rulings by Italian judges have defined, with ever-increasing clarity, just how much or how little information should be offered to patients prior to obtaining their consent to any medical-surgical treatment. In the same way, successive Italian codes of medical ethics have increasingly highlighted the importance of information in any fruitful dialogue between physician and patient. Just what this information should contain has been and still is subject to many variables which, undoubtedly, closely reflect changing cultural values over the years.

Thus deontology codes have gone from favouring the exclusion of patients from the information flow in the event of an unfavourable prognosis – something which is hardly feasible nowadays but once in line with a paternalistic view of medicine – to awarding them absolute centrality and autonomy through information and dialogue instigated by the physician [2]. Finally, the code recently approved now stipulates that physicians have an ethical duty to inform the patient of each unwanted event and its causes, and to identify, report adverse events, near misses, procedural and diagnostic errors. Thus the obligation to supply information and to communicate continues to widen, in some way extending beyond the doctor-patient relationship to become an essential tool for improving the quality of professional services.

## Discussion

### Italian codes of medical ethics

Successive deontological codes in Italy from the 1970’s until today reflect profound changes in the doctor-patient relationship. This position is summed up and underlined in the statement, now consolidated in deontological standards, that ‘the physician must not undertake diagnostic and/or therapeutic activity without the acquisition of the patient’s explicit and informed consent’. It appears, therefore, that Italian deontological sensibility has by now fully understood and accepted the spirit of informed consent which expresses the essentiality and centrality of the patient’s autonomy in relation to the power of the physician, who is free to act only when the patient has expressed informed consent. It is not simply a matter of consent, therefore, but also of information. Deontological codes have awarded greater importance to information as an ineluctable part of consent. A question remaining open for Italian doctors concerns the amount of information to be provided to the patient, particularly regarding risks. This is a complex and frequently recurring problem in the medical profession, which Italian judges have sought to resolve by emitting a huge number of sentences with the aim of setting limits to information [3].

The very latest version of the code has nothing to add in this regard; thus it appears that the question of information limits remains open. For the first time, however, the code calls for ‘*honest and correct’* information and for communication of error and adverse event as a tool for controlling quality and managing clinical risk. These are new additions which help to develop a more open attitude with regard to information for the patient. Such an attitude is essential for improved clinical risk management; furthermore, it meets the patient’s expectations and is part of the wider responsibilities (including those of a managerial nature) involved in practising medicine. Finally, it is essential for the same principles of dignity and freedom underpinning the issue of informed consent.

It is clear that the new deontological precepts intersect two areas in which the figure of the physician is paramount. On the one hand is the need for maximum integrity towards the patient in interpersonal ethics, in the name of the doctor’s own, and the other’s (the patient’s) dignity and liberty; on the other is the physician’s developing role in the strategies of the health system to achieve efficacy, quality, reliability and efficiency, to reduce errors and to manage clinical risk.

### Honest and correct communication in the private physician-patient relationship

Communicating the truth to the patient is a crucial issue in the complex and demanding relationship between physician and patient. Current socio-cultural trends which in different ways condition the medical profession, as well as a greater focus on the dignity and rights of the patient, have led to a growing tendency to fully inform the patient with regard to his/her illness, progress and therapy. Nevertheless, it is still common for doctors to choose silence or to ‘manipulate’ the truth in some way, especially when prognosis is serious or negative and the patient seems to be having real difficulties accepting it.

The topic is a complex one, covering medical, psychological, legal and ethical aspects [4,5]. Many questions arise for the physician. What truth should be told? What methods and timing would be most appropriate and effective? Might silence not be better [6,7]?

The new code calls on the medical profession to act with integrity and reciprocal trust and to divulge honest and correct information. This would seem to convey an updated conception of the therapeutic relationship, in which truth is not just the transmission of scientifically precise information, but rather implies a more complex commitment to honesty on the part of the physician. Thus emerges a broader idea of truth and its place in the information flow. We believe that honest communication should not only entail the expression of scientifically correct information, which is undeniable, but that it must also consider the circumstances, time and place in the light of the existing relationship between the interlocutors. Clearly, then, lies are eschewed, but it also seems appropriate, in the extreme situation of difficult news, to take into account the overall level of truthfulness in the particular physician-patient relationship [8].

In the light of the stipulations of successive Italian deontological codes over the years, as well as the richness of the international debate on the subject, the new code suggests new guidelines for responsible communication of the truth to the patient. These take into account not only the scientific and technical truth of the information, but also the patient’s capacity to receive such information and the possible effects that the truth may have on that patient. The code of deontology seems to confirm the need for the physician’s continued intellectual efforts to first identify and evaluate the various elements of truth in question as precisely as possible, since truth is not always clearly defined within medical parameters and as regards the individual patient.

The concept of honest and correct communication is closely linked to that of the disclosure of adverse events and errors [9]. The latter, however, extends beyond truthfulness in the two -way physician-patient relationship and widens the scope of the deontological code to include the issues of patient safety and clinical risk management, in which the physician plays a role of primary importance.

### Honest and correct information as a tool in managing clinical risk

What it all comes down to is the exchange of truthful, correct information between physician and patient which includes communication of any untoward medical occurrence: adverse events (injuries that result from a medical intervention and are responsible for harm to the patient), near misses (adverse event that either resolves spontaneously or is neutralized by voluntary action before the consequences have time to develop) [10]. But honest, open and comprehensive communication surely implies the disclosure of procedural (non diagnostic) and diagnostic errors too [11]. As with adverse events which does not necessarily implies a personal medical liability, procedural and diagnostic errors, which for several branches of medicine has surpassed all other error categories, should be promptly disclosed to patients [12].

Communication of errors and adverse events, through an open, honest and transparent approach with the patient and his/her family is, in fact, fundamental to a relationship based on the reciprocal trust and loyalty which underpins the latest code of deontology.

There are a great many existing studies on full error disclosure and they depict a very varied landscape as well as grounds for applicability which are not always clear. Despite, in fact, a general willingness among physicians to admit their own mistakes to patients and their families, there is still a wide gap between theory and practice, because of a substantial divergence and irreconcilability of interests between the parties involved. Fear of being sued, the shame of error, and fear of economic consequences, may push physicians to conceal the unwanted event rather than reveal it. Fear of being held responsible for the unwanted event may affect the physician’s role with regard to communication, and fail to meet the patient’s expectations [13].

This is what emerges at an international level. There are no data to describe the Italian scenario; the only study of this kind conducted in Italy, in an Intensive Therapy Unit, and published in a European review [14], reveals that only 1.11% of doctors say they fully inform their patients (also about errors). On the other hand, 74%, while acknowledging their moral and ethical duty in such conduct, admit that they supply only a ‘sweetened’ version of the facts. These findings are in line with international studies. Many existing studies confirm a substantial gap between the physician’s willingness to disclose an error to the patient, and actual daily practice [15,16].

The occurrence of a medical error may be caused by a combination of human and system factors and it may be viewed in two ways: the individual and the system approach. Each has its model of error causation and each model gives rise to quite different philosophies of error management [17]. Medicine has traditionally treated errors as failings on the part of individual providers, reflecting inadequate knowledge or skill, so leading to a wide spread of the so called ‘name, blame and shame’ culture. The system approach, by contrast, takes the view that most errors reflect predictable human failings in the context of poorly designed systems. Rather than focusing corrective efforts on punishment or remediation, the system approach seeks to identify situations or factors likely to give rise to human error, and change the underlying systems of care in order to reduce the occurrence of errors or minimize their impact on patients [18,19]. Because healthcare-related harm is, very often, caused by system or process failures, it is important to adopt various process-improvement techniques to identify inefficiencies, ineffective care, and preventable errors to then influence changes associated with systems. A key factor is making any healthcare – related harm visible and easily disclosed.

Growing awareness of this issue has led some countries to issue laws making it obligatory for physicians to inform patients who are victims of adverse events or errors (for example, French law: loi Kouchner relative aux droits des malate et à la qualité du système de santé, 4 March 2002, no. 303). Moreover, in many countries the need of an open disclosure of adverse events and medical errors is felt at the institutional level [20,21].

In Italy, documents have been issued by the Ministry of Health [22] which emphasise that ‘Managing the relationship between health structures and patients on the occurrence of an adverse event demands a robust, clear, and well-defined approach. This should be based on a procedure shared by all the health structures of the National Health System regarding both the management of the adverse event as well as open and transparent communication with patients and their families about what has happened’. With a view to improving the quality of the health service and to managing clinical risk, the Ministry of Health specifies that ‘health workers have an ethical responsibility to maintain honest and transparent communication with patients and their families at all times during the healthcare process. This is particularly indispensable in the most difficult situations’.

The objectives are to guarantee the patient’s right to receive transparent and honest communication and, especially when there is an health care –related negative event, to favour accountability. The following are, in brief, the basic steps set out by the Ministry of Health. If the patient has been harmed by an event, it is necessary to explain, clearly and honestly, what has happened, and to supply adequate medical and psychological support. Errors and adverse events must be communicated to the patient by a health worker in the unit who knows the patient’s clinical history, and preferably by the referral physician. They must be communicated as soon as they happen, when the patient is clinically stable and able to understand what is said. If the adverse event has caused serious consequences, such as disability or death, family members or the patient’s legal representative must be informed quickly. As regards the manner of communication, an empathetic relationship should be established with the patient and/or family members. The physician must understand the mood and feelings of the patient and his/her background. A climate of honesty, transparency, participation and solidarity must be created, bearing in mind that all the people involved might be in an altered emotional state. The language used must be simple and easy to understand. Technical terms and jargon should be avoided as much as possible and all specialist terms should be explained in everyday language. It is best not to use the term ‘error’. It should be borne in mind that the culture of health and illness may vary according to the patient’s ethnic group, beliefs (also religious) and origins. The facts must be described clearly and unambiguously. The patient should be informed of the diagnostic-therapeutic-rehabilitative process, and be reassured that everything will be done to limit and mitigate the consequences. The patient should not be overwhelmed by an excess of information, but neither should the explanation be simplified to the extent that it is trivialized. The patient and/or family must be allowed sufficient time to assimilate the information received, and be invited to express any doubts and to ask questions.

The role of physicians becomes a part of a complex strategy of risk management based on a system focused approach whose objectives are to increase transparency regarding adverse outcomes and support physicians in disclosing adverse outcomes to patients, to investigate via root – causes analysis and explain what happened, to improve patient safety and implement systems to avoid recurrence of incidents, using information from cases of medical injury and near misses to identify safety-enhancing interventions and working with hospital staff to implement them. Finally, conciliation system through the healthcare institution’s insurance is implemented as a tool for alternative dispute resolution that may avoid lawsuits, reduce liability costs, and improve access to compensation by meeting the financial needs of injured patients and their families quickly and fairly in the aftermath of an injury.

In this context the role of healthcare givers is of paramount importance with regard to the informational burden. It could not be otherwise. It is up to the health care giver who is, in some way, involved in the negative event, to explain to the patient and relatives what happened. Educational programs implement trainings in disclosure skills are provided, and hospitals and supervising physicians provide and support training.

In this scenario, the provisions of the code of medical ethics could hardly remain insensitive to a growing need for quality in health services, involving the direct participation of the physician. The new code meets the need for patient safety by underlining the central role of the physician who must contribute to the prevention and management of clinical risk. This is achieved through good clinical practice and all other methods approved by the scientific community to individuate therapeutic treatments which are safe and consistent with need. However, it also depends on the physician’s utmost care to inform the patient and secure his/her consent, including the disclosure of an unwanted event and its causes, and to identify, report, evaluate, and assess the causes of sentinel events, errors, ‘near-misses’ and adverse events [23-25].

## Summary

The new elements regarding information in the Italian code of medical ethics would seem to depict a new kind of physician, called into question on the one hand for his/her communication skills (true scientific information, timing, methods, terms used), as well as his/her skill in involving the patient in the flow of information. On the other hand, the physician is obliged to personally guarantee the safety of the patient not only by supplying high quality medical care with good clinical practice and all the means approved by the scientific community, but also by informing the patient on adverse events and errors.

The topic of disclosure cannot be discussed without addressing the issue of its impact on professional liability exposure and fear of litigation [13,16]. How can a medical error or unanticipated outcome be openly disclosed to a patient or patient’s family without increasing the risk of litigation? How can a disclosure policy be successful when the healthcare providers’ fear of being sued for medical malpractice makes them view their patients only as plaintiffs? In a regional Italian survey of healthcare providers on their attitude to disclosing their own errors, up to 31% of the respondents said they do not report medical errors because of liability concerns. Physicians demonstrate particular reluctance to disclose information about a medical error for this reason [26].

This is an issue that should be seriously addressed in the development stage of a disclosure policy. Certainly, the deontological standards which provide for communication as a moment of ‘exclusive technical-professional reflection which is reserved and confidential and aims at correcting the procedure and modifying organizational practices and professional behaviour’ may not be sufficient to reassure physicians with regard to the risks of litigation.

## Competing interests

The authors declare that they have no competing interest.

## Authors’ contributions

All authors contributed to the paper. ET and MN contributed to conception and design of the article, drafted the article and all revised it critically for important intellectual content. Both authors read and approved the final manuscript.

## Authors’ information

ET is Full Professor and Chief of the Department of Legal Medicine, University of Foggia, Foggia, Italy.

MN is Aggregate Professor at the Department of Legal Medicine, University of Foggia, Foggia, Italy.

## Pre-publication history

The pre-publication history for this paper can be accessed here:

http://www.biomedcentral.com/1472-6939/15/57/prepub
